# Circulating levels of adropin and diabetes: a systematic review and meta-analysis of observational studies

**DOI:** 10.1186/s12902-023-01327-0

**Published:** 2023-04-07

**Authors:** Sepideh Soltani, Sara Beigrezaei, Mahsa Malekahmadi, Cain C. T. Clark, Shima Abdollahi

**Affiliations:** 1 Yazd Cardiovascular Research Center, Non-communicable Diseases Research Institute, Shahid Sadoughi University of Medical Sciences, Yazd, Iran; 2 Nutrition and Food Security Research Center, Shahid Sadoughi University of Medical Sciences, Yazd, Iran; 3 Department of Nutrition, School of Public Health, Shahid Sadoughi University of Medical Sciences, Yazd, Iran; 4 Research Center for Gastroenterology and Liver Disease, Shahid Beheshti University of Medical Sciences, Tehran, Iran; 5 Centre for Intelligent Healthcare, Coventry University, Coventry, CV1 5FB UK; 6 Department of Nutrition, School of Public Health, North Khorasan University of Medical Sciences, Bojnurd, Iran

**Keywords:** Adropin, Type 2 diabetes, Systematic review, Meta-analysis, Observational studies

## Abstract

**Objective:**

Adropin, a newly identified regulatory protein has garnered attention given its potential role in metabolism regulation, especially glucose metabolism and insulin resistance. However, studies on the association between adropin and type 2 diabetes mellitus (T2DM) are equivocal. The aim of this study is to assess the association between serum adropin levels and T2DM using a systematic review and meta-analysis of observational studies.

**Methods:**

PubMed, Scopus, ISI Web of science, and Google Scholar were searched, up to August 2022, for studies that reported the association between serum levels of adropin in adults with T2DM compared to a control group without diabetes. A random-effect model was used to compute the pooled weighted mean difference (WMD) with 95% confidence intervals (CI).

**Results:**

Meta-analysis of 15 studies (n = 2813 participants) revealed that the serum adropin concentrations were significantly lower in patients with T2DM compared with the control group (WMD= -0.60 ng/mL, 95% CI: -0.70 to -0.49; I^2^ = 99.5%). Subgroup analysis also found lower concentration of adropin in patients with T2DM who were otherwise healthy compared to a control group (n = 9; WMD=-0.04 ng/ml, 95% CI= -0.06 to -0.01, p = 0.002; I^2^ = 96.4).

**Conclusions:**

Our study showed adropin levels are lower in patients with diabetes compared to a control group without diabetes. However, the limitations of observational studies challenge the validity of the results, and further investigations are needed to confirm the veracity of these findings and additionally explore possible mechanisms.

## Introduction

Type 2 diabetes mellitus (T2DM) is a major global public health concern. Worldwide, the total number of people with T2DM has inexorably grown, and is estimated that, by 2040, its’ prevalence will reach approximately 570 million patients [1–3]. Patients with diabetes experience a wide range of complications, including micro and macro-vascular damages, leading to cardio-vascular diseases, neuropathy, retinopathy, or nephropathy [2, 4]. Given the progressive nature of the diabetes and no definitive cure for it, improved knowledge about the pathogenic pathways could lead to finding new biomarkers for early diagnostic and treatment development of diabetes.

Although the mechanisms underlying diabetes progression are not fully understood, it has been proposed that signaling molecules secreted in multiple tissues (e.g., hepatokines, myokines, adipokines) are involved [5]. One of these molecules, adropin, has recently attracted much attention. Adropin is a polypeptide of 76 amino acids encoded by the *Energy Homeostasis Associated* (*Enho*) gene, and Kumar et al., [6] initially identified its’ presence in the liver and brain of mouse models. Interestingly, in humans, adropin is also produced in the same amino acid sequence as mice, in the postrema and dorsal vagal complex areas in the brain and hepatocytes, which are involved in regulation of metabolism and energy hemostasis [6, 7]. Further, adropin has also observed in other areas of the central nervous system and other tissues, including kidney, lung, testes, gut, peripheral blood mononuclear cells, skeletal muscle, endothelial cells, and also is present in the circulatory system [6–9].

Although studies on adropin are still in their infancy, it has been proposed that adropin produces its multiple functions through interaction with the G protein-coupled receptor 19 (GPR19) [10], mainly in the brain, cardiac, some cancerous cells [10–12], and with Nb-3/Notch1 in the brain [13]. Adropin also can improve endothelial cell survival, and neovascularization in human umbilical vein and coronary artery endothelial cells, through modulation of vascular endothelial growth factor receptor-2 (VEGFR2), activation of the PI3K-Akt and ERK1/2 pathways, increased endothelial nitric oxide synthase bioactivity, and nitric oxide-dependent signaling pathways [14].

It is also proposed that adropin-deficient status may play a role in the pathogenesis of diabetes. Studies have reported that adropin-knockout mice showed an increase in adipose tissue and impaired glucose metabolism [15], and adropin treatment of diet-induced obese mice improved glucose metabolism and insulin signaling [6, 16, 17]. Although the exact mechanism has remained poorly defined, one of the underlying mechanisms appears to be associated with downregulation of *pyruvate dehydrogenase kinase-4* (*PDH kinase-4*) expression, which leads to inhibition of fatty acid oxidation and higher glucose oxidation, due to increased activity of pyruvate dehydrogenase, an enzymatic complex involved in acetyl-coenzyme A formation; so it can enter into the citric acid cycle [18]. Moreover, Gao et al. suggested that insulin-induced cell surface expression of glucose transpoter-4 (GLUT4) gene expression is increased by adropin, especially in muscle cells, and is related to improved glucose uptake [16]. Concomitantly, carnitine palmitoyltransferase-1B and CD36, the two critical enzymes in fatty acid metabolism, are inhibited by adropin, reducing fatty acid uptake, and β-oxidation in mouse muscle cells [18]. As fatty acid over load is associated with insulin resistance [19], the regulatory role of adropin in switching the energy substrate from fatty acid to glucose may be contributory to the improvement insulin resistance. Further evidence indicates that adropin can suppress adipogenesis by inhibiting differentiation of 3T3-L1 cells to the mature adipocytes through GPR19 receptor, and ERK1/2 and AKT dependent mechanisms [20, 21]. Moreover, it is well known that obesity is related to increased cellular inflammation. The endoplasmic reticulum is one of the organelles affected by stress, and its homeostasis is disturbed, and, in turn, hepatic c-Jun N-terminal kinase activity increases, leading to phosphorylation of insulin receptor, and suppression of insulin signaling [22]. A recent study found that adropin treatment reduced endoplasmic reticulum stress responses and reduced hepatic c-Jun N-terminal kinase activity, which improved insulin signaling pathways in the liver, consequently [23].

There are a few observational studies showing that adropin levels are reduced in patients with T2DM [15, 24], and women with polycystic ovary syndrome (PCOS) [25, 26]. By contrast, some other studies have reported that serum levels of adropin may increase in patients with T2DM [27, 28]. Given the previous contradictory results, we aimed to investigate the association between adropin levels in patients with T2DM, compared with a control group without diabetes, by performing a systematic review and meta-analysis of observational studies.

## Methods

This systematic review and meta-analysis was conducted based on the PRISMA statement (Preferred Reporting Items for Systematic Reviews and Meta-Analyses) [29].

### Search strategy

Articles were obtained through a comprehensive systematic electronic search of PubMed, Scopus, Web of Science, and Google Scholar databases, up to 13th August 2022, using the term “adropin”. “Adropin” was searched in all fields of the electronic databases, without any date and language restriction, to comprehensively retrieve all the potentially relevant papers. Moreover, the reference lists of included studies were checked to find additional related articles. Two independent investigators screened articles (SS and MM), and any disagreement was resolved by consultation with a third investigator (SA).

### Eligibility criteria

Observational studies that reported the mean and corresponding variance of adropin serum concentrations in adults with T2DM in comparison with a reference group (without diabetes) were eligible for inclusion in this systematic review. The exclusion criteria were as follows: studies which were (a) case reports, editorials, reviews, and conference abstract; (b) conducted in children, pregnant/ lactating women; (c) carried out on cell lines or animal models; (d) enrolled non-diabetic participants, and/or gestational diabetes; and (e) without a reference group for comparison.

### Data extraction

Two investigators (SB, MM) extracted data from eligible studies, independently, and any discrepancy was resolved by discussion with the corresponding author. The following data were extracted from each eligible study: first author’s name, year of publication, country of origin, characteristics and sex of studied population, diagnostic criteria for diabetes, sample size, diabetes-related complications, comorbid diseases, methods of adropin measurement, mean and corresponding variance of adropin levels, and potential confounders.

### Quality assessment of articles

Quality of the studies was assessed by independent investigators according to the Newcastle-Ottawa Scale (NOS). The NOS tool evaluates the quality of included studies across seven items categorized in three domains: selection, comparability, and outcome with a maximum score of 10 [30]. Studies with a score ≥ 7 were assigned high quality, whereas those with score ≤ 5 were considered low quality.

### Statistical analysis

We used the means and their corresponding SD of serum adropin concentrations in cases with T2DM and their controls to calculate the effect size. The weighted mean differences (WMD), with the corresponding 95% CI, were pooled using a random effects model to take into account the variability between true study effects [31]. Cochran’s Q test and I^2^ were used to assess statistical heterogeneity [32]. Subgroup analyses for study location, diabetes-related complications, and adjustment of confounders were performed to identify the possible sources of heterogeneity. Sensitivity analysis was conducted to describe the effect of individual studies on pooled analysis. Funnel plots and Egger’s tests were used to examine the publication bias [33]. Statistical analyses were performed by STATA version 17 (STATA Corp., College Station, TX, USA), and P values less than 0.05 were considered statistically significant.

## Results

**Fig. 1:**
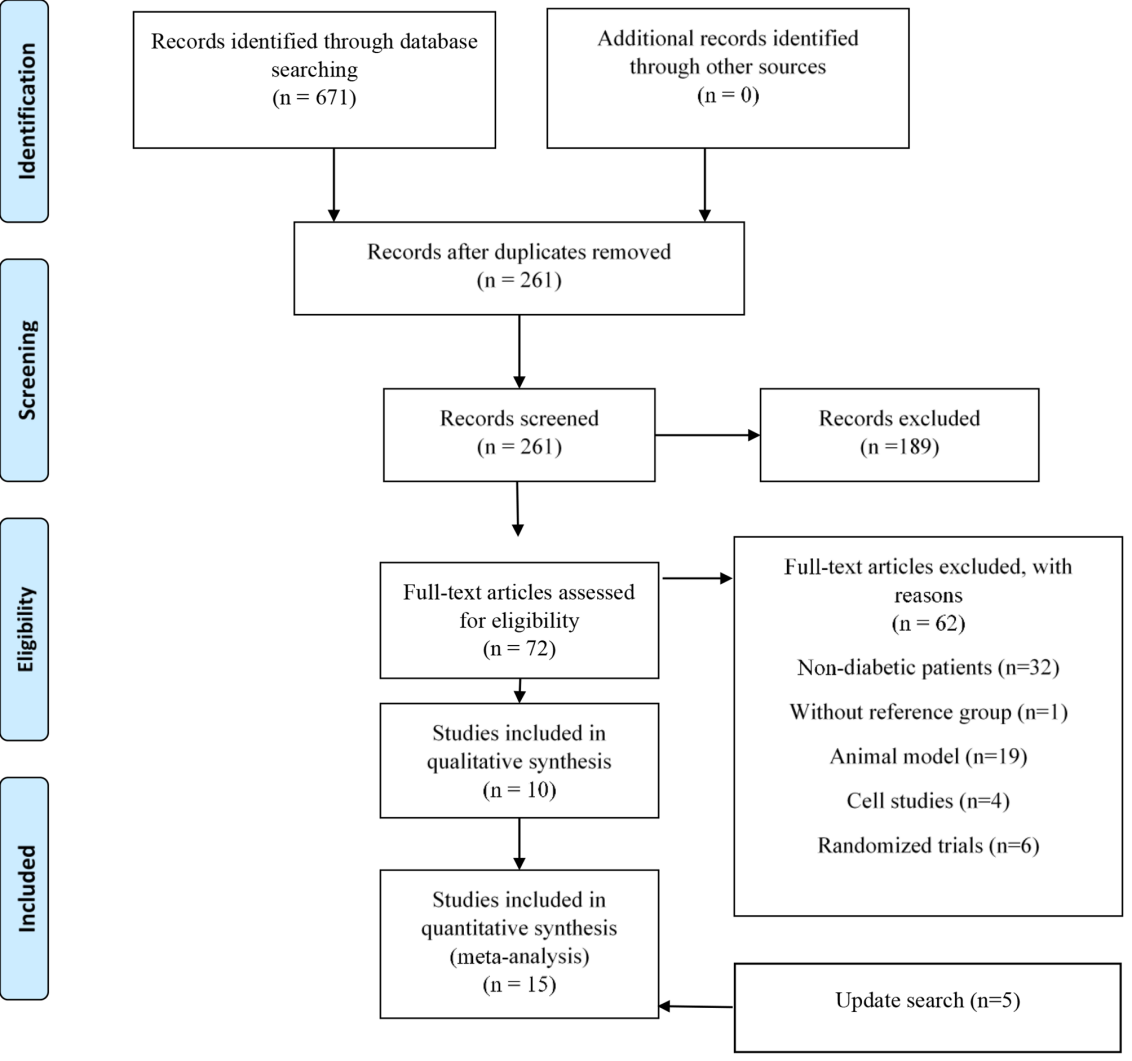
Flowchart of the selection of studies for the systematic review and meta-analysis

Of the 671 retrieved studies, 261 remained after duplicates removed, and 72 remained after title/abstract screening. A total of 62 studies were excluded after screening the full-texts for the following reasons: non-diabetic patients (n = 32) [25, 34–64], without reference group (n = 1) [17], animal model (n = 19) [7, 23, 65–81], cell studies (n = 4) [12, 82–84], and randomized trials (n = 6) [85–90]. Update search on the 13th of August 2022, yielded additional five eligible studies. Finally, 15 relevant articles, involving 2813 subjects, were included in the current meta-analysis (Fig. 1).

### Study characteristics

Table 1 summarizes the characteristics of the 15 eligible studies [24, 27, 91–103]. These studies were published between 2014 and 2022, and conducted in China (n = 7) [24, 91, 93, 94, 98, 101, 103], Iran (n = 3) [27, 92, 100], Turkey (n = 2) [95, 99], Iraq (n = 1) [96], India (n = 1) [102], and Ukraine (n = 1) [97]. Of all included studies, four studies were performed using a case-control design [27, 92, 95, 96], and eleven had a cross-sectional design [24, 91, 93, 94, 97–103]. Thirteen studies enrolled both male and female participants [24, 91–95, 97–103]; while two studies exclusively included females [96] or males [27]. The participants of four studies had diabetes-related complications, including albuminuria [93, 94], retinopathy [91, 93], and hypertension [97]. The method of serum adropin measurement was ELIZA in all the included studies [24, 27, 91–103]. The diagnostic criteria for T2DM was based on the American Diabetes Association (ADA) in eight studies [24, 27, 91–94, 98, 102], European Association for the Study of Diabetes (EASD) in one study [97], HOMA-IR cut-off in one study [101], and World Health Organization (WHO) in another study [93]. The remaining five studies did not provide information about the diagnosis criteria [95, 96, 99, 100, 103]. The confounding factors that were examined in the analyses included age and sex [24, 91, 93, 94, 98], BMI [24, 93, 98], lipid profile [27, 93, 98], and blood pressure values [93, 98]. However, nine studies reported non-adjusted association [92, 95–97, 99–103].

**Table 1:** The main characteristic of included observational studies

First Author (Publication year)	Country/ Study design	Sex/ Sample size	Comorbid chronic disease	Method of measurement	Diagnostic criteria for T2DM/ Diabetes-related complications	Confounders	Adropin levels (Mean ± SD) ng/mL	
							Cases	Controls
Addai et al. (2018) [96]	Iraq/ CC	F / 30	PCOS	ELISA	HOMA - IR > 2.7/ None	Not adjusted	0.14 ± 0.01	0.14 ± 0.01
Aydın et al. (2022) [99]	Turkey/ CS	B/ 50	None	ELISA	NM/ None	Not adjusted	0.26 ± 0.07	0.64 ± 0.39
Eshaghi et al. (2021) [100]	Iran/ CS	B/ 134	None	ELISA	NM/ None	Not adjusted	3.06 ± 0.46	4.21 ± 0.52
Hosseini et al. (2016) [27]	Iran/ CC	M / 80	None	ELISA	ADA/ None	WHR, FBG, LDL-C, HOMA-IR, HbA1c	2.5 ± 1.92	1.9 ± 1.46
Hu et al. (2018) [94]	China/ CS	B / 571	None	ELISA	ADA/ Albuminuria	Sex and age	2.71 ± 0.7	3.7 ± 1.31
Kutlu et al. (2019) [95]	Turkey/ CC	B / 81	NAFLD	ELISA	HOMA - IR > 2.7/ None	Not adjusted	0.6 ± 0.2	0.77 ± 0.32
Li, B et al. (2020) [93]	China/ CS	B / 447	None	ELISA	ADA and WHO/ Albuminuria and retinopathy	Sex, age, BMI, duration of diabetes, SBP, DBP, TC, TG, LDL-C, HbA1c, HOMA-IR, CP, NEP and CHIT1	0.16 ± 0.01^*^	0.16 ± 0.01
Li, N et al. (2021) [101]	China/ CS	B / 30	NAFLD	ELISA	HOMA - IR > 2.7/ None	Not adjusted	0.51 ± 0.73	4 ± 3.52
Li, S et al. (2019) [91]	China/ CS	B / 233	None	ELISA	ADA/ Retinopathy	Sex and age	2.5 ± 0.6	3.84 ± 1.48
Palizban et al. (2019) [92]	Iran/ CC	B / 146	None	ELISA	ADA/ None	Not adjusted	12.32 ± 2.98	9.51 ± 2.73
Shah et al. (2021) [102]	India/ CS	B/ 100	None	ELISA	ADA/ None	Not adjusted	2.12 ± 0.12	3.82 ± 0.4
Shelest et al. (2018) [97]	Ukraine/ CS	B / 119	Hypertension and Obesity	ELISA	EASD/ Hypertension and obesity	Not adjusted	2.74 ± 0.78	4.9 ± 0.58
Wu et al. (2014) [24]	China/ CS	B / 392	Suspected coronary artery disease	ELISA	ADA/ Suspected coronary artery disease	Sex, age, BMI	4.8 ± 1.48	5.53 ± 1.62
Zang et al. (2018) [98]	China/ CS	B / 176	None	ELISA	ADA/ None	Sex, age, BMI, smoking and drinking, SBP, DBP, TC, TG, LDL-C, HDL-C, hs-CRP	4.1 ± 1.88	5.7 ± 3.2
Zhang et al. (2020) [103]	China/ CS	B/ 224	None	ELISA	None/ retinopathy	Not adjusted	0.012 ± 0.011	0.18 ± 0.008

*The combined effect size is presented.

ADA, American Diabetes Association; B, Both; BMI, Body mass index; CC, Case-control study; CHIT1, Chitotriosidase; CP, Copeptin; CS, Cross- sectional study; DBP, Diastolic blood pressure; EASD, European Association for the Study of Diabetes; F, Female; FBG, Fasting blood glucose; HbA1c, Glycosylated hemoglobin A1c; HDL-C, High density lipoprotein cholesterol; HbA1c, Glycosylated hemoglobin A1c; hs-CRP, High sensitivity c-reactive protein; LDL-C, Low density lipoprotein cholesterol; M, Male; NAFLD, Nonalcoholic fatty liver disease; NEP, neprilysin; NM, Not mentioned; PCOS, Poly cystic ovarian syndrome; SBP, Systolic blood pressure; SD, Standard deviation; T2DM, Type 2 diabetes mellitus; TC, Total cholesterol; TG, Triglyceride; WHO, World Health Organization.

The results of quality assessment, based on the Newcastle-Ottawa scale, suggested that the six studies had good methodological quality [24, 27, 91, 93, 94, 98], whilst others were of moderate [92, 97, 99–103], and low quality [95, 96], respectively (Table 2). Except for four studies [96, 97, 99, 101], all the included studies used a sample that was representative of the average of our interested population. However, no description was provided about the characteristics of the responders and the non-responders across all studies. Another source of bias was related to the confounding factors, which were not controlled for [92, 95–97, 99–103], or a few number of possible confounding factors were considered, in the analyses [27]. Finally, no details were provided for the assessment of outcome in two studies [95, 96], and no score was assigned for this domain.

**Table 2:** Quality assessment of included studies, using New-Castle Ottawa scale

Author	Selection Bias				Comparability	Outcome		Score	Quality
	Representativeness of sample	Sample size	Non-respondents	Ascertainment exposure	Comparability	Assessment outcome	Statistical test		
Addai et al. (2018) [96]	--	--	--	**	--	--	*	3	Low
Aydın et al. (2022) [99]	--	--	--	**	--	**	*	5	Moderate
Eshaghi et al. (2021) [100]	*	--	--	**	--	**	*	6	Moderate
Hosseini et al. (2016) [27]	*	--	--	**	*	**	*	7	Good
Hu et al. (2018) [94]	*	*	--	**	**	**	*	9	Good
Kutlu et al. (2019) [95]	*	--	--	**	--	--	*	4	Low
Li, B et al. (2020) [93]	*	--	--	**	**	**	*	8	Good
Li, N et al. (2021) [101]	--	--	--	**	--	**	*	5	Moderate
Li, S et al. (2019) [91]	*	--	--	**	**	**	*	8	Good
Palizban et al. (2019) [92]	*	--	--	**	--	**	*	6	Moderate
Shah et al. (2021) [102]	*	--	--	**	--	**	*	6	Moderate
Shelest et al. (2018) [97]	--	--	--	**	--	**	*	5	Moderate
Wu et al. (2014) [24]	*	--	--	**	**	**	*	8	Good
Zang et al. (2018) [98]	*	--	--	**	**	**	*	8	Good
Zhang et al. (2020) [103]	*	--	--	**	--	**	*	6	Moderate

### Meta-analysis

**Fig. 2:**
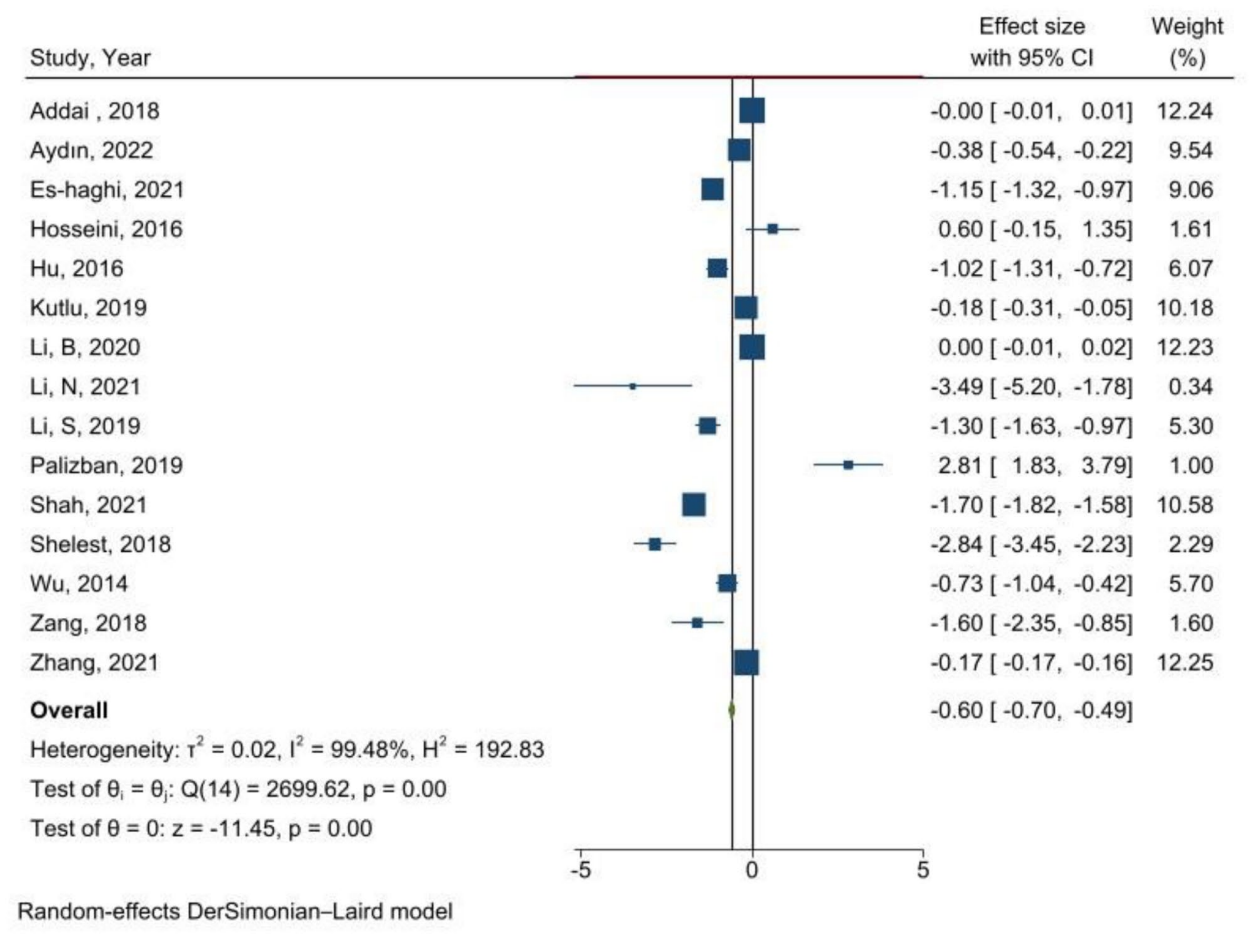
Forest plot demonstrating meta-analysis of studies evaluating adropin levels in T2DM participants compared to control group. (The analysis was done using random effects model. P-value of effect < 0.05)

Overall, meta-analysis of 15 studies [24, 27, 91–103] (n = 2,813 participants) revealed that the serum levels of adropin were significantly lower in patients with T2DM compared with non-diabetic participants (WMD= -0.60 ng/mL, 95% CI: -0.70 to -0.49, P < 0.001; I^2^ = 99.48%, P-heterogeneity < 0.001) (Fig. 2). A high between-study heterogeneity was observed, of unknown origin, even after subgroup analysis. The subgroup analysis indicated lower levels of adropin in patients with T2DM otherwise healthy (n = 9; WMD= -0.60 ng/mL, 95% CI: -0.70 to -0.49; I^2^ = 99.5%). Moreover, a lower adropin levels was observed in patients with T2DM in sex and age-adjusted studies (n = 5; WMD=-0.90 ng/ml, 95% CI= -0.70 to -0.49, p < 0.001; I^2^ = 97.2), compared to the studies did not adjust for these confounders (n = 10; WMD= -0.58 ng/ml, 95% CI= -0.71 to -0.45, p < 0.001; I^2^ = 99.55). Moreover, pooling data of BMI-adjusted studies failed to show a significant association between adropin levels and T2DM (n = 3; WMD= -0.69 ng/ml, 95% CI= -1.45 to 0.06, p < 0.001; I^2^ = 94.8), this is while, non-adjusted associations showed a significant lower level of adropin in patients with T2DM compared to the control group (n = 12; WMD= -0.67 ng/ml, 95% CI= -0.79 to -0.54, p < 0.001; I^2^ = 99.5) (Table 3).

**Table 3:** Meta-analysis evaluating the association between serum adropin levels and T2DM based on several subgroups and the overall analysis. (All analysis was done using random effects model. P-value of effect < 0.05)

Study group	Number of Studies	Meta-analysis		Heterogeneity			
		WMD (95%CI)	P effect	Q statistic	P within group	I^2^ (%)	P between group
**Overall**	15	-0.60 (-0.70 to -0.49)	< 0.001	2699.62	< 0.001	99.5	
**Study location**							
Asia	14	-0.54 (-0.64 to -0.44)	< 0.001	2624.06	< 0.001	99.5	< 0.001
Europe	1	-2.84 (-3.45 to -2.23)	< 0.001	0.00	-	-	
**Diabetes complications**							0.08
None (healthy participants)	9	-0.04 (-0.06 to -0.01)	0.002	223.11	< 0.001	96.4	
Microvascular complications	5	-0.83 (-1.70 to 0.05)	0.064	338.84	< 0.001	98.8	
Metabolic disorders^*^	3	-0.18 (-0.48, 0.11)	0.22	22.8	< 0.001	91.2	
Macrovascular complications	2	-1.76 (-3.82, 0.3)	0.09	36.64	< 0.001	97.3	
**Age and sex adjustment**							
Yes	5	-0.90 (-0.70 to -0.49)	0.010	143.03	< 0.001	97.2	0.372
No	10	-0.58 (-0.71 to -0.45)	< 0.001	1949.42	< 0.001	99.55	
**BMI adjustment**							< 0.001
Yes	3	-0.69 (-1.45 to 0.06)	0.072	38.63	< 0.001	94.8	
No	12	-0.67 (-0.79 to -0.54)	< 0.001	2027.83	< 0.001	99.5	

^*^Including poly cystic overran syndrome and non−alcoholic fatty liver

### Sensitivity analysis and publication bias

Findings from sensitivity analysis indicated results remained stable after removal of each study, individually, from the analysis.

As assessed by visual inspection, no asymmetry in the funnel plots was identified. Likewise, no significant publication bias was detected using asymmetry tests for meta-analysis (Begg’s test, P = 0.839; Egger’s test, P = 0.217).

## Discussion

The main finding of this meta-analysis indicated a significantly lower adropin concentration in patients with T2DM compared with a non-diabetic control group. This association was more pronounced when the analysis was adjusted for age and sex in original studies.

In support of our finding, evidence from animal models demonstrated a decrease in hepatic gluconeogenic regulatory enzymes, blood glucose levels, HOMA-IR, and a marked increase in serum insulin levels and insulin sensitivity index following adropin treatment [7, 18, 104, 105].

These beneficial effects of adropin have been explained via sensitization of insulin signaling pathways, and improved glucose oxidation. Adropin seems to increase insulin-mediated AKT phosphorylation and augments GLUT4 cell-surface expression in muscle cells, leading to glucose oxidation in these cells [16]. Furthermore, adropin is proposed to downregulate pyruvate dehydrogenase kinase gene expression (*PDH kinase-4*), and subsequently reduce its inhibitory effect on pyruvate dehydrogenase complex, and increased glycolysis [16]. Another recent study reported enhanced AKT signaling pathway, and suppression activity of FoxO1 and GSK3 by adropin treatment, resulting to a reduction in hepatic glucose production, and increased glycogen synthesis [23]. However, in the present study, we found significantly lower levels of adropin in patients with diabetes. As a possible justification, some studies reported increased adropin levels following hyperglycemia [7, 81, 105, 106], and since none of the included studies examined newly diagnosed patients, the observed decreased adropin levels may be due to damage to the organs responsible for the production of adropin, which is also targeted for diabetes, such as the pancreas or liver.

A reduction in adropin levels in obesity may also have contributed to the observed results [26, 98, 101, 107]. Although not all studies reported the body weight of participants, this justification is not implausible, given the high-prevalence of obesity in patients with T2DM. In support of this hypothesis, the association between lower levels of adropin and T2DM disappeared when our analysis was restricted to BMI-adjusted associations. Although it is not yet well understood how adropin levels decrease in the presence of obesity, it has been shown that adropin-knockout mice exhibit development of insulin resistance and metabolic dysregulation, and treatment with exogenous adropin, may improve these conditions [15]. Ghoshal et al., [108] also suggested a complex age-dependent association between adropin levels and obesity indices. Another study also found lower levels of adropin concentrations in obese patients compared to non-obese healthy individuals [109]. However, it must be acknowledged that some studies did not observe any significant association between obesity and adropin levels [107, 110]. Our recent meta-analysis on the adropin levels and obesity found a mean value of adropin levels in obese patients 2.01 ± 1.29 ng/mL, which was significantly lower compared to non-obese participants (WMD = − 0.96 ng/ml, 95% CI = − 1.72 to − 0.19, P = 0.01) [111]. However, in the current study, we found a relatively higher concentration of adropin in patients with T2DM (2.58 ± 0.8 ng/mL); which may be contributory to the low number of included studies in our previous study (n = 5), and a wide interval of reported values, which may not be a true representation of the obese population.

As another explanation, some studies have shown an inverse association between inflammation and adropin levels [112, 113]. As a chronic low-grade inflammation has been observed in diabetes, it may reduce the expression of the *Enho* gene in this situation. Akcilar et al. [104] also confirm this hypothesis, and showed an increase in TNF-α and IL-6 in diabetes-induced rats, which decreased after intraperitoneal adropin application. Additionally, Wu et al. [24] found a significant increase of hs-CRP in patients with diabetes in comparison to control group, and also an inverse association between adropin and hs-CRP levels.

Our results also showed that age and sex may affect adropin levels, which was in line with some previous studies [9, 108]. Although the mechanisms are not completely clear, it may be that sex-dependent hormonal differences and age-related metabolism changes contributed. Moreover, this can indirectly indicate an association between adiposity and adropin levels, which is affected by age and sex. Further studies evaluating these confounding factors are required to discern the precise mechanisms for this interaction.

The current systematic review and meta-analysis is, to date, the first comprehensive analysis to evaluate the association between serum adropin concentration and T2DM. Nevertheless, there are some potential limitations. Our meta-analysis is based on observational studies, which questions the validity of the results. Observational evidence is known to be to prone confounding bias, as several important characteristics may not be adjusted in the studies; for instance, in the present study weight of participants, medicines and treatment approaches, dietary intake, physical activity, duration of T2DM history, body composition etc., were not considered in original studies. Moreover, adropin gene expression in liver is thought to be affected by circadian rhythm, macronutrient intake, and weight changes. However, it is not clear whether studies took these confounding issues into account, which may bias our results. In addition, included studies did not provide sufficient data to perform additional analysis for the association between serum adropin and indices of glucose homeostasis. On the other hand, different populations, settings, and adjustment methods between studies may produce a high heterogeneity when pooling observational evidence, as we also observed an unknown source of heterogeneity between studies. It should also be noted we pooled together results of adropin that were measured either in serum or plasma, which may be the source of heterogeneity. Inclusion criteria, and matching process are also often less restrictive in observational studies than randomized trials [114]. Furthermore, observational evidence is prone to presence of selection bias since no randomization is performed, and therefore they cannot provide any casual inferences. Despite the aforementioned limitations, meta-analysis of observational analysis can be useful when clinical data is not sufficient. Finally, all included studies, except one, were conducted in Asian nations, which may have reduced the generalizability of our results, rendering them non-applicable, or of limited applicability, to other populations. Overall, the interpretation made from the findings of this meta-analysis should be taken cautiously.

## Conclusion

In conclusion, our meta-analysis demonstrated that there is an inverse association between adropin levels and T2DM; it seems that adropin might be a viable candidate for eliciting a protective effect against T2DM. However, this finding was manifest from a low number of studies which could decrease the robustness of our findings. More investigations are still needed to clarify the molecular mechanisms that justify this association in different geographical zones.

## Data Availability

The datasets used and/or analysed during the current study are available from the corresponding author on reasonable request.
